# Airway oxidative stress in chronic cough

**DOI:** 10.1186/1745-9974-9-26

**Published:** 2013-12-02

**Authors:** Heikki O Koskela, Minna K Purokivi

**Affiliations:** 1 Unit for Medicine and Clinical Research, Pulmonary Division, Kuopio University Hospital, PL 1777, 70211 Kuopio, Finland

**Keywords:** Chronic cough, Cough, Cough provocation tests, Oxidative stress, Isoprostanes

## Abstract

**Background:**

The mechanisms of chronic cough are unclear. Many reactive oxygen species affect airway sensory C-fibres which are capable to induce cough. Several chronic lung diseases are characterised by cough and oxidative stress. In asthma, an association between the cough severity and airway oxidative stress has been demonstrated. The present study was conducted to investigate whether airway oxidative stress is associated with chronic cough in subjects without chronic lung diseases.

**Methods:**

Exhaled breath condensate samples were obtained in 43 non-smoking patients with chronic cough and 15 healthy subjects. Exclusion criteria included a doctor’s diagnosis of any lung disorders and any abnormality in lung x-ray. The concentration of 8-isoprostane was measured. In addition, the patients filled in Leicester Cough Questionnaire and underwent hypertonic saline cough provocation test, spirometry, ambulatory peak flow monitoring, nitric oxide measurement, and histamine airway challenge. In a subgroup of patients the measurements were repeated during 12 weeks’ treatment with inhaled budesonide, 800 ug/day.

**Results:**

The 8-isoprostane concentrations were higher in the cough patients than in the healthy subjects (24.6 ± 1.2 pg/ml *vs.* 10.1 ± 1.7 pg/ml, p = 0.045). The 8-isoprostane concentration was associated with the Leicester Cough Questionnaire total score (p = 0.044) but not with the cough sensitivity to saline or other tests. Budesonide treatment did not affect the 8-isoprostane concentrations.

**Conclusions:**

Chronic cough seems to be associated with airway oxidative stress in subjects with chronic cough but without chronic lung diseases. This finding may help to develop novel antitussive drugs.

**Trial registration:**

The study was registered in ClinicalTrials.gov database (KUH5801112), identifier NCT00859274.

## Background

Chronic cough is reported by 10 – 20% of general population [1]. It is the most common condition for which patients seek consultation from a doctor [2]. The mechanisms of chronic cough are poorly understood. As a consequence, there are very few therapeutic options for patients with unexplained chronic cough. There is a pressing need to understand the genetic, molecular, and physiological basis of chronic cough and to develop novel antitussive drugs [3].

During the recent years the concept of chronic cough as a sensory neuropathy has been widely accepted [4]. C-fibres represent one of the two main sensory pathways to initiate cough reflex and are often thought to be the principal fibres mediating cough in pathological conditions [5]. Many reactive oxygen (ROS) and nitrogen (RNS) species activate sensory bronchopulmonary C- fibres, probably through Transient Receptor Potential A1 channels [6-8]. Peroxidation of arachidonic acid by ROS leads to formation of isoprostanes [9]. Animal studies show that isoprostanes sensitise C-fibres in a concentration-dependent manner [10].

An imbalance between the manifestation of ROS and an organ’s ability to detoxify them is called the oxidative stress. Many chronic lung diseases including asthma, chronic obstructive pulmonary disease, cystic fibrosis, and fibrosing lung diseases are characterised by oxidative stress [11-17]. Cough is a typical symptom of all these disorders. In asthma, high exhaled breath condensate (EBC) 8-isoprostane levels are associated with poor cough-associated quality of life and hypersensitivity to the cough-provoking effect of hyperpnoea [18]. Thus, airway oxidative stress may be involved in the pathogenesis of cough in chronic lung diseases, probably by affecting the function of sensory bronchopulmonary C- fibres. However, in the majority of patients with chronic cough there is no evidence of chronic lung diseases. The present study was planned to investigate whether airway oxidative stress is present also in subjects without doctor’s diagnosis of any chronic lung disorders but who seek medical advice due to chronic cough.

## Methods

### Subjects

Forty-three subjects with chronic cough of at least eight weeks’ duration were recruited using newspaper advertisements. Exclusion criteria were a doctor’s diagnosis of asthma at present or previously, doctor’s diagnosis of any other lung disorder, current smoking, any abnormalities in chest x-ray, and a febrile respiratory tract infection within six weeks. In addition, 15 healthy, non-smoking subjects were recruited from the personnel of the authors’ department. Table 1 shows the characteristics of the subjects.

**Table 1:** The basic characteristics of the subjects

**Characteristic**	**Subjects with chronic cough (N = 43)**	**Healthy subjects (N = 15)**
Age (years)	55.6 ± 1.9	52.7 ± 4.1
Females – number (%)	32 (74)	11 (73)
Ex smokers – number (%)	20 (47)	4 (27)
Atopic patients – number (%)	14 (33)	1 (7)
Duration of cough (years)	8.5 ± 1.5	ND
Most probable cause of cough^a^,	Rhinitis 22 (51)	ND
- number (%)	Esophageal reflux 14 (33)	
	Asthma 9 (21)	
Leicester questionnaire total score	13.2 ± 0.5	ND
Cough sensitivity to hypertonic saline (coughs/Osmol/kg)	7.24 ± 1.03	ND
Histamine PD_15_ (mg)^b^	1.28 ± 1.20	ND
Nitric oxide concentration (ppm)	16.8 ± 2.0	ND
FEV_1_ (% of predicted)	93.7 ± 1.8	ND
PEF variation in ambulatory monitoring (%)	7.13 ± 0.68	ND

Continuous data is expressed as means and standard errors. ND = not done; FEV_1_:= forced expiratory volume in one second; PD_15_ = provocative dose of histamine to induce a 15% fall in FEV_1_. PEF = peak expiratory flow.

^a^Based on the results of the Cough Clinic diagnostic questionnaire. Two patients had more than one most probable cause.

^b^Among subjects without a 15% fall in FEV_1_ in response to histamine, an arbitrary value of 3.2 mg was used as PD_15_. Histamine challenge was missed in one patient due to technical reasons.

The study was performed in accordance with the Good Clinical Practice guidelines recommended by the Declaration of Helsinki. The study was approved by the institutional Ethics Committee (132//2008) as well as the National Agency for Medicines (EudraCT 2009-009556-21). The study was registered in ClinicalTrials.gov database (KUH5801112), identifier NCT00859274. Written informed consent was obtained from each subject prior to participation in the study.

### Study design

**Figure 1:**
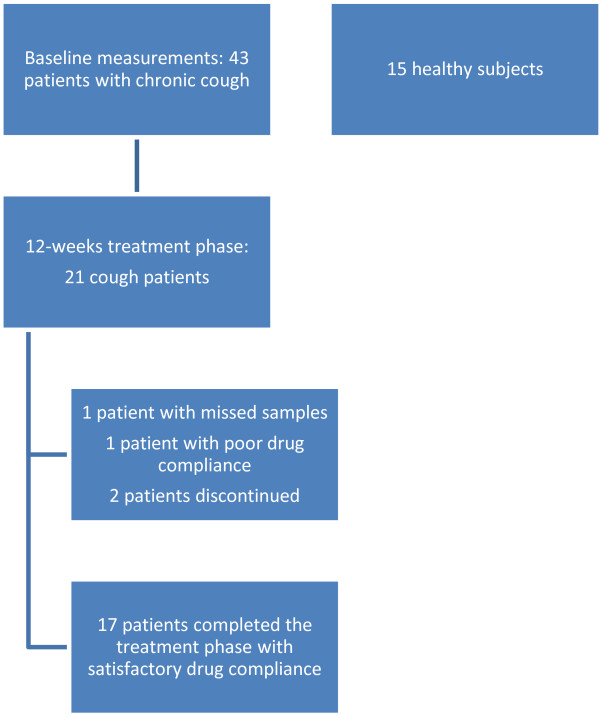
The subject flow chart.

This was a prospective study including a wide range of baseline measurements and an open-label treatment phase in a subgroup of cough patients (Figure 1). Its methods and clinical results have been published previously [19]. The present paper describes the results of the EBC analysis of 8-isoprostane.

At baseline, the patients with chronic cough underwent the following tests and measurements: The Leicester Cough Questionnaire (LCQ) [20] and The Cough Clinic diagnostic questionnaire [21] were filled in. A chest x-ray, skin prick tests, exhaled air nitric oxide (NO) measurement, ambulatory peak expiratory flow (PEF) monitoring, histamine airway challenge [22], spirometry, and hypertonic saline cough provocation test [23] were performed. Histamine challenge and the saline test were performed on separate days. In the cough patients, the first EBC was collected after the spirometry, before the saline test, and the second two minutes after the saline test. The healthy subjects did not perform the spirometry and the saline test but only underwent the EBC sample collection.

The treatment phase was participated by the cough patients who were responsive to the hypertonic saline test (N = 21). For the next twelve weeks, they used budesonide inhalation powder 400 ug twice a day (Budesonid Easyhaler, Orion Ltd, Espoo, Finland). The EBC samples were collected and the Leicester Cough Questionnaire was filled in at one, four, and twelve weeks. In one subject the baseline EBC sample was missed due to technical reasons, two subjects did not complete the treatment phase and one was found to have non-satisfactory drug compliance.

### Leicester Cough Questionnaire

The Leicester Cough Questionnaire is a 19-item validated, repeatable and responsive questionnaire consisting of physical, psychological and social domains. Answers are graded on 7-point Likert scale which gives a total score ranging from 3 to 21. A small score indicates poor cough-related quality of life [20].

### The Cough Clinic diagnostic questionnaire

The Cough Clinic diagnostic questionnaire includes 16 questions designed to differentiate the three common causes of chronic cough (gastro-oesophageal reflux disease, asthma, and rhinitis). The three components of the questionnaire are validated but the whole questionnaire and algorithm is not [21]. Idiopathic chronic cough cannot be detected by this questionnaire.

### Hypertonic saline cough provocation test

First, spirometry was performed for three times. The subjects inhaled 0.4 mg of salbutamol to prevent bronchoconstriction. Fifteen minutes after the salbutamol inhalation the spirometry was repeated. Then the subject inhaled isotonic phosphate-buffered saline for two minutes via a high-output ultrasonic nebuliser (DeVilbiss Ultraneb 3000, Sunrise Medical Ltd, West Midlands, UK), using tidal breathing. The coughs occurring during the inhalation and two minutes after it were counted up. The number of these “spontaneous” coughs was subtracted from the coughs provoked by each hypertonic solution. Subsequently, they inhaled hypertonic phosphate-buffered saline solutions with osmolalities of 600, 900, 1200, 1500, 1800 and 2100 mOsm/kg. The challenge was stopped if the subject asked for it or if 15 or more cumulative coughs were recorded. Two minutes after the final saline solution the spirometry was repeated [23].

The cough sensitivity to hypertonic saline was expressed as the cumulative number of provoked coughs divided by the final osmolality inhaled (CDRsaline). The unit of this index is coughs/Osmol/kg.

### Collection and analysis of the EBC

EBC was collected according to published guidelines [24] utilizing a commercially-available device (Ecoscreen Turbo, VIASYS Healthcare GmbH, Höchberg, Germany). The device has a salivary trap to avoid salivary contamination of the samples. The subjects sat and wore a nose clip. The duration of collection was 10 minutes, using tidal breathing. The condensate was stored in – 70°C and analysed afterwards. The 8-isoprostane concentrations were measured by enzyme immunoassay utilizing a commercial reagent according to the manufacturer’s instructions (Cayman Chemical Company, Ann Arbor, MI, USA). Samples were diluted directly in EIA Buffer without purification. A standard curve was established by dilution of the 8-isoprostane EIA Standard between 1500 pg/ml and 2.5 pg/ml, using EIA Buffer as the matrix.

Altogether, 217 EBC samples were obtained during the study. In 38 samples (18%) the 8-isoprostane concentrations were below the detection limit of the assay. In these samples, an arbitrary value of 1.25 pg/ml was utilized for statistical purposes. Baseline EBC sample was missed in one subject due to technical reasons.

### Statistical analysis

The continuous data is expressed as means and standard errors. The distributions of the EBC 8-isoprostane concentrations and histamine airway responsiveness differed significantly from the normal distribution (p < 0.05, Kolmogorov-Smirnov test). However, these values were normally distributed after logarithmic transformation. Therefore, log-transformed values were used for parametric statistics and geometric means and standard errors were expressed. T-tests, repeated measures analysis of variance, Pearson correlation coefficient (Rp), and analysis of covariance with backwards directed stepwise procedure were utilized when appropriate.

## Results

**Figure 2:**
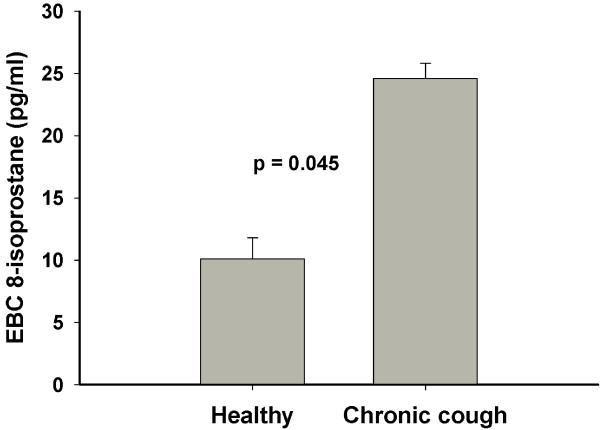
Comparison of the exhaled breath condensate (EBC) 8-isoprostane concentrations between 15 healthy subjects and 43 patients with chronic cough.

The baseline, pre-saline EBC 8-isoprostane concentrations were higher in the cough patients than in the healthy subjects (24.6 ± 1.2 pg/ml vs. 10.1 ± 1.7 pg/ml, p = 0.045, Figure 2). According to the Cough Clinic diagnostic questionnaire, the most probable underlying condition among the cough patients were rhinitis (22 patients) esophageal reflux (14 patients), and asthma (9 patients). In two patients the questionnaire suggested more than one underlying condition. Exclusion of the nine possible asthmatic patients from analysis did not affect these results significantly: The corresponding 8-isoprostane concentrations were 24.0 ± 1.2 pg/ml in the cough patients and 10.1 ± 1.7 pg/ml in the healthy subjects ( p = 0.063).

**Figure 3:**
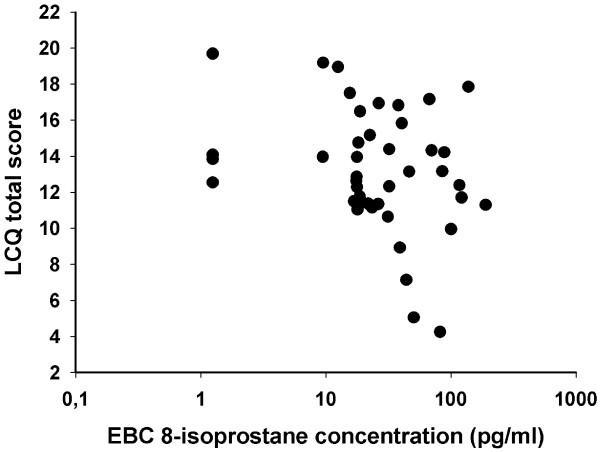
**Association of the exhaled breath condensate (EBC) 8-isoprostane concentration and Leicester Cough Questionnaire (LCQ) total score among 43 patients with chronic cough (Rp = -0.29, p = 0.06).** Low LCQ total score indicates poor cough-related quality of life.

**Figure 4:**
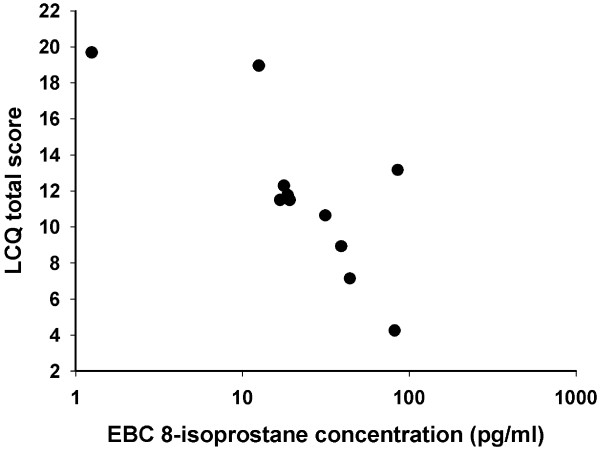
**Association of the exhaled breath condensate (EBC) 8-isoprostane concentration and Leicester Cough Questionnaire (LCQ) total score among the 11 male patients with chronic cough (Rp = -0.77, p = 0.006).** Low LCQ total score indicates poor cough-related quality of life.

Within the cough patients, a suggestive association between the baseline EBC 8-isoprostane concentration and the LCQ total score was found in the bivariate analysis (Rp = -0.29, p = 0.06, Figure 3). This association was much closer in men than in women (Rp = -0.77, p = 0.006 *vs.* Rp = -0.11, p = 0.54, respectively, Figure 4). When sex was included in the multivariate analysis, the association between baseline EBC 8-isoprostane concentration and the LCQ total score was statistically significant within the whole group of cough patients (p = 0.044).

The baseline EBC 8-isoprostane concentration did not correlate statistically significantly with PEF variation in ambulatory monitoring, spirometric indices, NO level, histamine airway responsiveness, and hypertonic saline cough responsiveness. The mean 8-Isoprostane concentrations did not differ between men and women (22.2 ± 1.4 *vs.* 25.6 ± 1.3 pg/ml, respectively (p = 0.75)). Furthermore, there were no differences in EBC 8-isoprostane concentrations between the patient groups divided according to the most probable underlying cause of chronic cough.

The 12-week budesonide treatment did not affect the EBC 8-isoprostane concentrations significantly (p = 0.39). There was no association between the baseline EBC 8-isoprostane and the maximal improvement in LCQ total score during the budesonide treatment (Rp = 0.06, p = 0.70).

The saline test did not affect the EBC 8-isoprostane concentrations: There were 100 pairs of EBC samples collected before and after the saline test. The 8-isoprostane concentrations were 17.9 ± 1.1 pg/ml and 16.6 ± 1.2 pg/ml (p = 0.56). The intraclass correlation coefficient between the pre- and post-saline measurements was 0.72.

## Discussion

The present study shows that airway oxidative stress may be associated with chronic cough among non-smoking patients without a doctor’s diagnosis of any chronic lung diseases. The evidence for this was twofold: First, the concentrations of EBC 8-isoprostane were higher in the patients with chronic cough than in the healthy subjects. Second, the EBC 8-isoprostane concentrations were associated with the Leicester Cough Questionnaire total scores within the cough patients.

The study population was recruited utilising a newspaper advertisement and probably well represents typical patients seeking help for chronic cough of unknown origin. The three most common underlying disorders among such patients are gastro-oesophageal reflux disease, asthma, and rhinitis [25]. Though a doctor’s diagnosis of any chronic lung disease was used as an exclusion criterion, nine of the present patients may have suffered from asthma, according to the Cough Clinic diagnostic questionnaire. It is well known that asthma is characterised by oxidative stress [13]. However, exclusion of the probable asthma patients from analysis did not affect the results significantly. Furthermore, there were no differences in EBC 8-isoprostane concentrations between the patient groups divided according to the most probable underlying cause of chronic cough. We acknowledge that the study group was too small for a subgroup analysis. However, these findings suggest that cough itself is the phenomenon associated with oxidative stress, not the underlying disorder. This is in close accordance with the new concept of cough as a sensory neuropathy [4].

Measurement of F(2)-isoprostanes is considered as the most reliable approach to assess oxidative stress status in vivo [26]. F(2) isoprostanes are stable compounds and relatively abundant in all normal biologic fluids and tissues. The most prevalent isoprostane in humans is 8-epi-PGF_2α_, also known as 8-isoprostane [27,28]. The presently utilized EIA kit to analyse 8-isoprostane is widely used in EBC samples [18,29-40], is validated against mass spectrometry [38], and has acceptable repeatability [38,41]. The short-term repeatability was satisfactory also in the present study when comparing the pre- and post-saline measurements. However, the present study suffers from similar difficulties to all studies utilising EBC samples. EBC is a very dilute solution of biomarkers and for many of them, assays are often employed at or near their detection limits [24]. This was also the case in the present study with 18% of the samples being below the detection limit of the 8-isoprostane assay. Therefore, the results should be interpreted cautiously.

As mentioned in the introduction, there is solid experimental evidence suggesting that airway oxidative stress might be involved in chronic cough with many ROS and RNS and their metabolites being capable to affect the function of sensory bronchopulmonary C-fibres [6-8,10]. The sources of ROS and RNS in the airways can be both endogenous and exogenous [42]. The exogenous sources of ROS and RNS are various environmental pollutants, especially airborne particulate matter and cigarette smoke [43-46]. The main endogenous ROS and RNS in the lung come from inflammatory cells like neutrophils, eosinophils, and alveolar macrophages, but also from structural cells [47]. This may explain the presence of oxidative stress in many chronic inflammatory lung diseases [13,15-17].

Various inflammatory cells have also been found in the airways of patients with chronic cough [48-50]. A lymphocytic airway inflammation has been identified in some patients with idiopathic chronic cough and organ specific autoimmune diseases [51]. In chronic cough, inflammation may be a consequence of mechanical trauma of coughing [52]. This might explain the facts that oxidative stress could be demonstrated in this kind of unselected cough population and that there were no differences in EBC 8-isoprostane concentrations between the subgroups with different possible causes of cough.

If inflammation is a consequence of coughing, oxidative stress might be the key factor to initiate a vicious circle of chronic cough: The original trigger may provoke cough that causes traumatic inflammation in the airways [52]. The inflammatory cells, in turn, generate ROS and RNS [47] which act on sensory C-fibres and thus induce further cough and secondary traumatic inflammation. By this means cough could continue even though the original trigger for cough may have disappeared long ago.

The association between 8-isoprostane and Leicester Cough Questionnaire total score was much closer in men than in women. This is difficult to explain. The mean 8-Isoprostane concentrations did not differ between the sexes. Epidemiological studies usually report similar prevalence of chronic cough in men and women [53]. However, data of specialist cough clinics clearly show that women more often than men seek medical advice due to cough [54]. Women were also overrepresented in the present study which recruited the subjects utilising newspaper advertisements. This may indicate that there are differences how men and women experience cough and might partly explain the finding of the present study.

In the present study population cough responsiveness to hypertonic saline did not correlate with the EBC 8-isoprostane concentration. On the contrary, a statistically significant correlation between the two biomarkers has been demonstrated among asthmatic patients. Furthermore, no association could be seen between cough responsiveness to hypertonic saline and LCQ total score in the present study. Again, a very close association between the two has been demonstrated in asthmatic patients [55]. These findings suggest that hypertonic saline cough provocation test reflects the cough severity better in asthma than in non-specific chronic cough.

None of the established asthma biomarkers namely ambulatory PEF variation, spirometric indices, NO level, and airway histamine responsiveness correlated statistically significantly with EBC 8-isoprostane. This suggests that the pathogenesis of airway oxidative stress in chronic cough is different from asthma pathogenesis.

The present study as well as previous studies [31,41,56,57] consistently show that the effect of inhaled corticosteroids on EBC 8-isoprostane concentrations is negligible. The previous follow-up studies about this issue may be criticized on the basis of short duration of corticosteroid treatment (3 – 4 weeks) but the present study shows that even a 12 weeks’ treatment with inhaled corticosteroid does not affect EBC 8-isoprostane concentrations in a statistically significant manner. Given that EBC 8-isoprostane analysis is considered as a reliable approach to assess airway oxidative stress status [27,28] it seems that inhaled corticosteroids have minor effect on airway oxidative stress. This finding may explain the poor effect of inhaled corticosteroids in many patients with chronic cough. The beneficial effect of corticosteroids in certain patients with chronic cough [19,58,59] is probably mediated through suppression of airway eosinophilic inflammation [49,60].

This study may be criticised by the fact that EBC was collected after the spirometry in the cough patients whereas in the healthy subjects spirometry was not performed. To the best of the authors’ knowledge, there are no studies about the effect of forced expiratory manoeuvres on EBC 8-isoprostane concentration. If such an effect does exist, it may have affected the described difference in EBC 8-isoprostane concentration between the cough patients and the healthy subjects.

## Conclusions

The results of the present study, together with the earlier one among asthmatic patients [18], suggest that airway oxidative stress may be involved in the pathogenesis of chronic cough. Airway oxidative stress may be the common factor behind cough in various pulmonary and extrapulmonary disorders and could, at least partly, clarify the mechanisms of sensory neuropathy in chronic cough [4]. Further studies aiming at assessing oxidative stress in subjects with chronic cough using independent non-invasive techniques, including e-noses [61,62] and nuclear magnetic resonance spectroscopy of EBC [63,64], should be undertaken. The inability of inhaled corticosteroids to suppress airway oxidative stress may explain the often unsatisfactory effect of these drugs on chronic cough. The present findings may help to develop novel antitussive drugs. Inhaled antioxidants might be an interesting treatment option to be evaluated in chronic cough.

## Abbreviations

CDR: Coughs-to-dose ratio; EBC: Exhaled air condensate; LCQ: Leicester Cough Questionnaire; NO: Exhaled air nitric oxide concentration; PEF: Peak expiratory flow; RNS: Reactive nitrogen species; ROS: Reactive oxygen species; Rp: Pearson correlation coefficient.

## Competing interests

Heikki Koskela owns Orion Ltd, Finland, shares worth 9000 euros. Minna Purokivi has no competing interests.

## Authors’ contributions

HK mainly designed the study, analyzed and interpreted the data, and wrote the manuscript. MP helped to design the study, partly analysed the data, and revised the manuscript critically for important intellectual content. Both authors have read and approved the final version of the manuscript.
